# High Color Purity Lead‐Free Perovskite Light‐Emitting Diodes via Sn Stabilization

**DOI:** 10.1002/advs.201903213

**Published:** 2020-03-01

**Authors:** Hongyan Liang, Fanglong Yuan, Andrew Johnston, Congcong Gao, Hitarth Choubisa, Yuan Gao, Ya‐Kun Wang, Laxmi Kishore Sagar, Bin Sun, Peicheng Li, Golam Bappi, Bin Chen, Jun Li, Yunkun Wang, Yitong Dong, Dongxin Ma, Yunan Gao, Yongchang Liu, Mingjian Yuan, Makhsud I. Saidaminov, Sjoerd Hoogland, Zheng‐Hong Lu, Edward H. Sargent

**Affiliations:** ^1^ School of Materials Science and Engineering Tianjin University Tianjin 300350 P. R. China; ^2^ Department of Materials Science and Engineering University of Toronto Toronto ON M5S 3E4 Canada; ^3^ Department of Electrical and Computer Engineering University of Toronto Toronto ON M5S 3G4 Canada; ^4^ State Key Lab for Artificial Microstructure and Mesoscopic Physics School of Physics Peking University Beijing 100871 China; ^5^ Key Laboratory of Advanced Energy Materials Chemistry (Ministry of Education) College of Chemistry Nankai University Tianjin 300071 China

**Keywords:** antioxidation, H_3_PO_2_ additives, lead‐free perovskites, Sn‐based red light‐emitting diodes, Sn stabilization

## Abstract

Perovskite‐based light‐emitting diodes (PeLEDs) are now approaching the upper limits of external quantum efficiency (EQE); however, their application is currently limited by reliance on lead and by inadequate color purity. The Rec. 2020 requires Commission Internationale de l'Eclairage coordinates of (0.708, 0.292) for red emitters, but present‐day perovskite devices only achieve (0.71, 0.28). Here, lead‐free PeLEDs are reported with color coordinates of (0.706, 0.294)—the highest purity reported among red PeLEDs. The variation of the emission spectrum is also evaluated as a function of temperature and applied potential, finding that emission redshifts by <3 nm under low temperature and by <0.3 nm V^−1^ with operating voltage. The prominent oxidation pathway of Sn is identified and this is suppressed with the aid of H_3_PO_2_. This strategy prevents the oxidation of the constituent precursors, through both its moderate reducing properties and through its forming complexes with the perovskite that increase the energetic barrier toward Sn oxidation. The H_3_PO_2_ additionally seeds crystal growth during film formation, improving film quality. PeLEDs are reported with an EQE of 0.3% and a brightness of 70 cd m^−2^; this is the record among reported red‐emitting, lead‐free PeLEDs.

## Graphical abstract

Sn‐based perovskite light‐emitting diodes with ultra‐high red color purity, a brightness of 70 cd m^−2^, and 24 nm linewidth are prepared. The devices show excellent color stability under different temperatures, power, and operating voltage. Based on the oxidation pathway of Sn, H_3_PO_2_ is chosen to suppress the oxidation of Sn^2+^ and slow down the crystal growth, simultaneously.

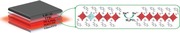

Metal halide perovskites have attracted intense interest in recent years as promising candidates for next‐generation displays.^[^ ^1^, ^2^ ^]^ In particular, perovskite‐based light‐emitting diodes (PeLEDs) with superior external quantum efficiency (EQE) and luminance have been demonstrated in the green, red, and near‐infrared emission regions.^[^ ^3^, ^4^, ^5^, ^6^ ^]^ As display technologies continue to improve, the requirements for the emitters in the display become more stringent. As of August 2012, the Rec. 2020 standard defines the display color gamut for ultrahigh definition television (UHDTV): it requires each of the primary red, green, blue (RGB) emitters to have a precisely defined wavelength (red: 630 nm, green: 532 nm, and blue: 467 nm) and a narrow emission linewidth (<20 nm).^[^ ^7^ ^]^ In state‐of‐art liquid crystal display backlights, crosstalk between color filters reduces the color purity of the RGB primaries.^[^ ^8^ ^]^

LED displays do not require color filters and as such are promising candidate for UHDTV. Organic molecules have had success in commercial LED displays, but the wide linewidths (>50 nm) of organic emitters limit the attainable color gamut.^[^ ^9^ ^]^ Developing emitters with precisely defined emission profiles for displays is an active area of research; next‐generation emitters such as colloidal chalcogenide quantum dots (QDs) and metal halide perovskites are of particular interest due to their narrow emission linewidth and tunable spectra. Cd‐based QDs have demonstrated the necessary emission linewidths and central emission frequencies, but their commercial application is limited by the toxicity of the cadmium.^[^ ^10^, ^11^, ^12^ ^]^

Similarly, while metal halide perovskites—particularly the reduced dimensional variant, in which a bulky organic cation is incorporated into the framework of the perovskite—have demonstrated excellent color purity and efficiency for green emitters, they have until now relied on the use of lead.^[^ ^13^ ^]^ Red and blue perovskite emitters also require partial halide substitution to tune the emission spectra to the wavelengths; but the mobile halogen ions undergo phase segregation, producing a subsequent shift in emission during operation, compromising color stability.^[^ ^14^, ^15^ ^]^ There is thus significant interest in developing Pb‐free perovskites, ideally composed of a single halide.

Replacing Pb with a divalent cation such as Sn has had success when employed in perovskite solar cells.^[^ ^16^, ^17^ ^]^ In particular, the 2D phenethylammonium tin iodide (PEA_2_SnI_4_, PSI) perovskite has emission centered at 633 nm, ideal for red emitters.^[^ ^18^ ^]^ However, Sn‐based perovskites suffer ready oxidation of Sn^2+^ to Sn^4+^, leaving undesired Sn^2+^ vacancies that act as nonradiative recombination centers and quench emission.^[^ ^19^, ^20^ ^]^ As a result, there is thus far only one report on PSI LEDs: the device exhibited a low luminance of 0.15 cd m^−2^, with an EQE that was not measurable.^[^ ^21^ ^]^

The oxidation of Sn^2+^ must be suppressed to realize efficient LEDs; previously, SnF_2_ had been widely used as a Sn compensator to mitigate Sn oxidation in solar cells.^[^ ^22^, ^23^, ^24^, ^25^ ^]^ However, the SnF_2_ causes phase separation—its incorporation into the film is inhomogeneous, and this results in poor film quality and low device performance.^[^ ^26^ ^]^ Reducing agents such as hydrazine (N_2_H_4_) vapor have been used to reduce the environmental oxygen concentration during film formation and to reduce Sn^4+^ to Sn^2+^, but the volatility of hydrazine can result in a local over‐reduction of Sn^2+^ to metallic Sn, which also functions as a nonradiative recombination center.^[^ ^17^ ^]^ Gentler reducing agents such as ascorbic acid and Sn powder have also been used to retard the oxidation of Sn, but they are solid and difficult to remove from the final perovskite film, which might harm the film quality.^[^ ^27^, ^28^ ^]^ The role each of the aforementioned dopants was to provide a reducing atmosphere; what requires further consideration is the interaction of the dopant and the perovskite. In addition, the oxidation pathway is different for different types of Sn‐perovskite, and as a result the same reducing agent will have a different efficacy when employed in different systems.

We took the view that once the oxidation pathway of Sn^2+^ had been identified in PSI, we would be able to select a judiciously chosen reducing agent that would suppress oxidation without compromising film quality. We determined through thermogravimetric analysis (TGA) that a necessary first step in the oxidation of Sn^2+^ is the formation of SnI_4_. This led us to incorporate the liquid reducing agent, H_3_PO_2_ (HPA), to provide reducing conditions and also to inhibit the formation of the SnI_4_ complex. HPA has been reported as an additive in bulk Sn‐perovskite solar cells and forms intermediate complexes with the perovskite during film formation:^[^ ^26^ ^]^ these intermediate complexes prevent the formation of SnI_4_ and thus retard the oxidation of Sn^2+^ in PSI. Additionally, the complexes seed crystal growth and improve the final quality of the film, in contrast with the inhomogeneous solid reducing agents that have been used to date. Here we combine HPA with 2D Sn‐based perovskites to achieve a record brightness and EQE for red lead‐free PeLEDs.

The resultant Pb‐free PeLEDs exhibit stable emission centered at 633 nm with a narrow full width at half maximum (FWHM) of 24 nm; the Commission Internationale de l'Eclairage (CIE) *x*, *y* coordinates of the PeLEDs are (0.706, 0.294), matching closely with the Rec. 2020 red standard of (0.708, 0.292). The PeLEDs achieve a maximum luminance of 70 cd m^−2^, more than two orders of magnitude higher than the best previously reported red Pb‐free PeLEDs. This advance is obtained only as a result of the reduced trap state density and improved film quality due to HPA incorporation. The high luminance and excellent color coordinate make this LED a candidate for next‐generation displays.

We chose to investigate 2D‐iodide based Sn‐perovskites, PSI, since the quantum well formed by the bulky organic ligands leads to an emission profile centered at 633 nm. In contrast to Pb‐based perovskites used for red emitters, there is no need to mix halides to obtain the desired bandgap: avoiding the need for such a mix allows us to circumvent the problem of halide segregation and subsequent color‐shifting during LED operation.

The structure of the perovskite is shown in **Figure** **1**a. The absorption spectrum exhibits a sharp excitonic feature at ≈612 nm. X‐ray diffraction (XRD) data and grazing‐incidence wide‐angle X‐ray scattering confirm the layered structure and reveal that the quantum wells orient parallel to the surface: the strong (00l) reflections are aligned along the *q_z_* direction (Figure S1, Supporting Information). We then investigated the photoluminescence (PL) stability of PSI under different conditions. We measured the PL spectra of PSI at different temperatures: when the temperature was decreased from 290 to 20 K, there was minimal shift in the PL peak position (redshift ≈3 nm) (Figure 1c) and the FWHM was reduced by ≈6 nm (90–74 meV) (Figure 1d). The PL intensity only increased ≈2.5x at 20 K; this improvement is less than in bulk perovskites,^[^ ^29^ ^]^ an observation we attribute to the high exciton binding energy of 2D perovskites. The PL spectrum was also power invariant, as shown in Figure 1e,f and Figure S2 in the Supporting Information. Pesudo‐color plots of time‐resolved PL (TRPL) spectra in Figure 1 show that when the excitation density increased, the emission spectra are stable and the PL kinetics do not change.

**Figure 1:**
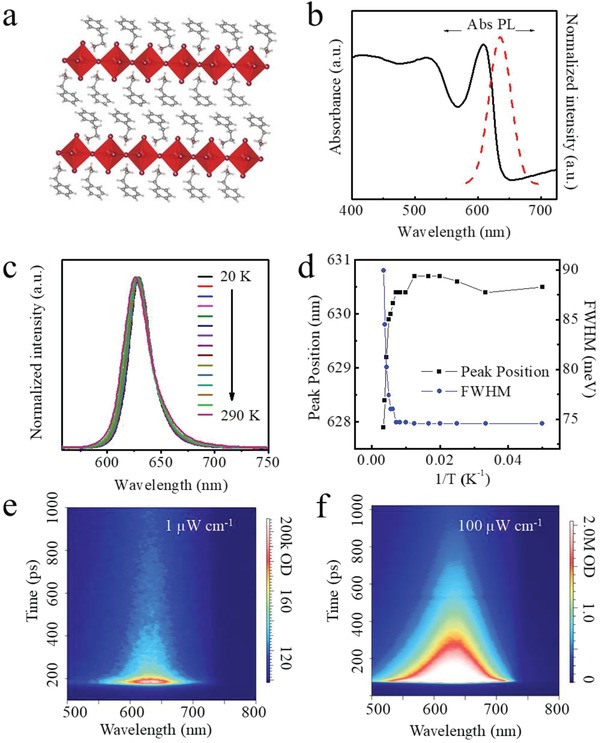
Structure and optical properties of PEA_2_SnI_4_ perovskite. a) Crystal schematic structure. b) UV–vis and PL spectra of PEA_2_SnI_4_ perovskite. c) Normalized PL spectra at various temperature from 20 to 290 K. d) PL peak position and FWHM versus 1/*T* data. e,f) Power‐dependent TRPL measurements pumped with 1 and 100 µW, respectively.

Although PSI has high color purity and color stability, the Sn^2+^ in Sn‐based perovskites is readily oxidized to Sn^4+^, resulting in Sn^2+^ vacancies that reduce the emission efficiency of the material.^[^ ^17^ ^]^ Mechanistic understanding of the oxidation process in Sn‐based perovskites can help enable an inhibition strategy. To identify whether oxidation was indeed occurring in the film, we carried out XRD on both fresh samples and on samples stored in air for one day. The main features in the XRD pattern of the oxidized sample mirror those of the fresh samples, with the exception of additional peaks, assigned to phenethylammonium iodide (PEAI), that are present in the oxidized sample (Figure S1a, Supporting Information). This result indicates that no phase transition occurred in the oxidized perovskite film, but that there was, in fact, partial degradation.

We sought to understand the process by which the degradation occurred: we used TGA to identify the degradation pathways as shown in Figure S3 in the Supporting Information. We identify two possible oxidation pathways in **Table** **1**.

**Table 1:** Oxidation pathways for the PSI and SnI_2_ precursor

	Mass loss	Final mass
PEA_2_SnI_4_		
(1) 2PEA_2_SnI_4_ + O_2_ → 4PEAI + SnO_2_ + SnI_4_(g)	35.3%	64.7%
(2) PEA_2_SnI_4_ + O_2_ → 2PEAI + SnO_2_ + I_2_(g)	14.3%	85.7%
Measured	35.1%	64.9%
SnI_2_		
(3) 2SnI_2_ + O_2_ → SnO_2_ + SnI_4_(g)	79.7%	20.3%
(4) SnI_2_+O_2_ → SnO_2_ + I_2_ (g)	59.5%	40.5%
Measured	79.6%	20.4%

In pathway **I**, as shown in Equations (1) and (3), Sn‐I bonds are partly broken between adjacent Sn octahedrons to produce equimolar SnI_4_ and SnO_2_ and in pathway **II** (Equations (2) and (4)) all Sn‐I bonds are broken and both Sn and I are oxidized to form SnO_2_ and I_2_.^[^ ^30^ ^]^ Both SnI_4_ and I_2_ can evaporate in the gaseous phase. These two pathways result in a different mass loss due to different gas contents and can thus be distinguished by TGA. We measured TGA on PEA_2_SnI_4_, SnI_4_, SnI_2_, and PEAI powders in N_2_ and also on PEA_2_SnI_4_ and SnI_2_ in air. We found that, when TGA was carried out in N_2_, the bulk of the mass loss of PEA_2_SnI_4_ and SnI_2_ occurred at higher temperatures than in air, indicating that the oxidation reaction is responsible for the products that are most readily vaporized. The TGA curves of PEA_2_SnI_4_ in air are characterized by two distinct mass losses, the first plateauing at 210 °C and accounting for 35.1% of mass loss and the second region of mass loss due to the evaporation of PEAI. The mass loss of SnI_2_ is 79.6% at 415 °C, due to the sublimation of SnI_4_.

We conclude that mass loss is due to the evaporation of SnI_4_ and that pathway **I** is the dominant one. In pathway **I**, fewer Sn‐I bonds are broken than in pathway **II**: the activation energy for oxidation pathway **I** is 537 meV, which is lower than that of the oxidation pathway **II** (731 meV) and is thus favored.^[^ ^30^ ^]^

After identifying the pathway of oxygen‐induced degradation of the Sn‐perovskite, we sought a strategy to prevent this degradation. From our analysis, a key component of the breakdown mechanism is the breaking of Sn‐I bonds. One way in which this dissociation can be inhibited is through complexes that limit the formation of SnI_4_. Previous reports added reducing agents that acted as oxygen scavengers, such as N_2_H_4_ and Sn metallic powder, which can reduce Sn^4+^ to Sn^2+^, but did not prevent the breaking of Sn‐I bonds.^[^ ^17^, ^28^ ^]^ SnF_2_ has been widely employed as Sn compensator to reduce the concentration of Sn vacancies; however, its inhomogeneous dispersion results in pinholes in the final films that are detrimental to device performance.^[^ ^16^ ^]^ We thus turned to HPA, which is a liquid reducing agent that forms complexes with perovskites—our reasoning was that this may inhibit oxidation without compromising film quality.^[^ ^26^, ^31^ ^]^

We observed that, after we added HPA, the oxidation of the precursor solution was prevented (Figure S4, Supporting Information). We carried out density‐functional theory (DFT) calculations and discovered that, in the presence of dimethyl sulfoxide (DMSO), HPA is oxidized more readily than Sn^2+^ (Note S1, Supporting Information), indicating that the oxidation of Sn^2+^ is retarded when HPA is present in the precursor solution. After we added HPA to the oxidized precursor solution, the dark color the solution lightened to the yellow of the unoxidized precursor, indicating that Sn^4+^ is reduced to Sn^2+^ (Figure S4e, Supporting Information). In addition to acting as a controllable reducing agent, HPA also forms a complex with Sn to form Sn(H_2_PO_2_)_2_, disrupting the adjacent Sn‐I octahedron. In the favored oxidation pathway, pathway **I**, the shared I ions transfer to an adjacent Sn cation to form SnI_4_. However, after the Sn‐HPA complex forms, the SnI_4_ cannot form as the Sn‐HPA complex inhibits the addition of more I ions.^[^ ^26^, ^31^ ^]^

We hypothesized that HPA reduces Sn^4+^, following a reaction mechanism with a reduction potential ≈0.65 V as shown in **Figure** **2**a. This was supported by a twofold improvement of the photoluminescence quantum yield (PLQY) (from 0.6% to 1.35%) of the final film when HPA was incorporated. We employed X‐ray photoelectron spectroscopy (XPS) to compare the Sn^4+^ content in the PSI films with and without HPA in the precursor solution. As shown in Figure 2b,c, we fit two peaks at 495.6 and 487.2 eV to each of the two samples; these were ascribed to the spin–orbit splitting of electrons from the 3d_3/2_ and 3d_5/2_ states of Sn^2+^, respectively. The additional two peaks at 496.6 and 488.2 eV were identified as the Sn 3d doublets corresponding to Sn^4+^ states.^[^ ^19^, ^26^ ^]^ The film with HPA additive has narrower Sn 3d peaks than that in the control film, and the peak area of Sn^4+^ is reduced. The elemental ratio of Sn^4+^:Sn^2+^ determined from the integrated peak areas decreases from 0.275 in the control film to 0.199 in the film with HPA additive, indicating the reducing additive retards the Sn^2+^ oxidation process. The appearance of P in XPS confirmed that trace amount of P still left in the PSI film, even after high temperature annealing (Figure S5, Supporting Information).

**Figure 2:**
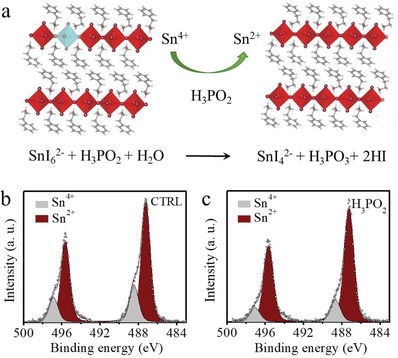
Role of HPA additive in preventing Sn^2+^ oxidation. a) Proposed mechanism of HPA reduction of Sn^4+^ to Sn^2+^. High‐resolution Sn 3d core level XPS spectra b) without or c) with HPA additive.

The inhibition of Sn^2+^ oxidation due to the reducing agent should result in a decreased in trap density; to compare relative trap densities, we employed transient absorption (TA) spectroscopy on films prepared with and without HPA additive. We first used a 400 nm pump pulse to excite the sample: for both samples the strongest induced photobleach was centered at 615 nm, in agreement with the steady‐state absorption spectra. The sample with the HPA additive shows a longer‐lived photobleached state than the control at the same power density (Figure S6, Supporting Information). The photobleaching intensity of both samples is similar when excited using a 400 nm pump pulse; photobleaching can occur both from trap‐state and band edge filling, and it is difficult to compare trap state densities using this excitation wavelength. We therefore used a 650 nm pump pulse to excite each film at sub‐bandgap energies; in this case electrons from the valence band can only be excited to mid‐gap states that act as trap states. As the electrons from the valence band occupy the mid‐gap states, there will still be a bleach signal observed at the band edge, as fewer electrons can now be excited from the valence band into the conduction band. In this way, sub‐bandgap narrowband TA offers a means to compare relative trap state densities when one normalizes the bleach signal to the steady‐state optical density of the sample.^[^ ^29^ ^]^ The pseudo‐color plots of the intensity change in the TA signal generated with a 650 nm pump, normalized to absorption (∆A/A) plotted against wavelength (nm) probe time delay (ps) are shown in **Figure** **3**a,b. The control film exhibits higher bleach intensity at 615 nm than the one with HPA treatment, (Figure 3d,e) with a faster decay rate as shown in Figure 3c. We also measured the PL decay (Figure 3f). The lifetime of the sample with the HPA additive is ≈1.1 ns, which is slower than that of the control sample 0.4 ns as shown in Table S1 in the Supporting Information. Taken together, these results indicate that the control film has a higher trap density than the sample prepared with the aid of HPA.

**Figure 3:**
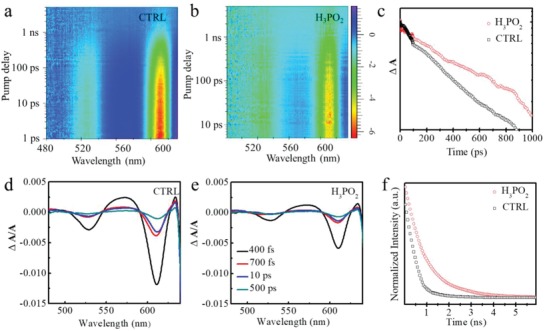
Photophysical studies of PSI films. a,b) TA plot. d,e) TA spectral at selected timescales with 650 nm laser pump of perovskite with or without HPA additive. c) TA signal at 615 nm with an excitation of 650 nm. f) PL time decay trace.

We also found that HPA does not compromise final film quality: the films are uniform (Figure S7, Supporting Information), with larger grain sizes and fewer grain boundaries. We attribute this to the ability of the intermediate complex that inhibits oxidation, Sn(H_2_PO_2_)_2_, to seed crystal growth of the film; the seeded growth serves as a means to grow larger crystal grains^[^ ^26^, ^31^ ^]^ (Figure S8, Supporting Information.) Intermediate complexes may also serve to passivate grain boundaries in the film, as shown in prior reports.^[^ ^27^ ^]^ The high film quality results in higher mobility (Figure S9, Supporting Information). HPA serves thus not only to reduce trap state density but also to improve final film quality through the formation of intermediate complexes; previously reported reducing agents also reduce trap state density, but leave detrimental residue in the final film, lowering the final film quality. We tested the optical stability of the film under continuous illumination (Figure S10, Supporting Information). The high color purity and improved PLQY of the HPA‐incorporating PSI films motivated us to explore PeLEDs. We fabricated devices via solution processing (Experimental Section). The energy level diagram and the configuration of the devices are shown in **Figure** **4**a,b. The valence band maximum energy is 5.23 eV, obtained by summing the work function and valence band maximum binding energy, both measured from ultraviolet photoelectron spectroscopy (UPS), as shown in Figure S11 in the Supporting Information. The conduction band minimum is obtained by adding the optical bandgap which is calculated from the edge of the absorption spectrum.

**Figure 4:**
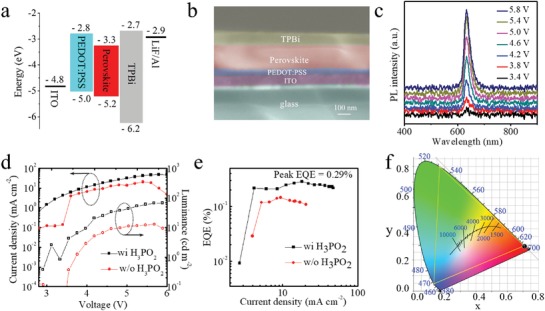
Device structure and optoelectronic characteristics of PeLEDs. a) Energy level diagram. b) Cross‐sectional SEM image of the PeLEDs. c) EL spectra of the device operating under different voltages. d) Dependence of the current density (left axis) and luminance (right axis) on driving voltage. Note that maximum luminance 70 cd m^−2^ of the device with H_3_PO_2_ is obtained under 5.8 V. e) EQE versus current density. A peak EQE of 0.3% is achieved at a current density of 18 mA m^−2^. f) The corresponding CIE coordinate of the device.

We employed a device architecture based on indium tin oxide (ITO)/poly(3,4‐ethylenedioxythiophene) polystyrenesulfonate (PEDOT:PSS) (≈30 nm)/PEA_2_SnI_4_ (≈200 nm)/TPBi (≈60 nm)/LiF (1 nm)/Al. Poly(2‐hydroxyethyl methacrylate) (PHM) was added to improve the film coverage and quality, a method previously reported to help maintain charge balance.^[^ ^6^ ^]^ We summarize device performance versus PHM additive in Figure S12 in the Supporting Information. The electroluminescence (EL) emission is centered at 633 nm with FWHM of 24 nm, shown in Figure 4c; the calculated CIE coordinates are (0.706, 0.294) (the ideal red color coordinate for Rec. 2020 is (0.708, 0.292)) as shown in Figure 4f. The color coordinates reported herein represent the highest color purity among red PeLEDs (Table S2, Supporting Information). The high color purity was maintained as a function of operating voltage, with excellent EL spectral stability as shown in Figure 4c.

Figure 4d shows the current density and luminance as a function of operating voltage for control samples and for the samples employing the HPA additive. In order to avoid over‐reduction of Sn^4+^ to form Sn^0^, we investigated a range of concentrations of HPA (Figure S13, Supporting Information). With the optimized HPA concentration, the maximum brightness of PeLEDs is 70 cd m^−2^ at a current density of 47.2 mA cm^−2^ under 5.8 V. This is ≈500 times higher than the best previous report of 2D‐Sn perovskites.^[^ ^21^ ^]^ The EQE is plotted as a function of current density in Figure 4e. The peak EQE of the device with the HPA additive is 0.3% with a current density of 18 mA cm^−2^, a factor of two times higher than that of the control sample. This represents the most efficient reported red lead‐free PeLED. Statistical data for device performance are shown in Figure S14 in the Supporting Information.

In summary, Sn‐based 2D perovskites were fabricated for ultrapure and stable red emission. LEDs incorporating the Sn‐based perovskites show EL emission centered at 633 nm with an FWHM of only 24 nm and CIE coordinates of (0.706, 0.294). These CIE coordinates are the most pure‐red among all PeLEDs. The optimized PeLEDs show a maximum luminance of 70 cd m^−2^ under 5.8 V, giving an EQE of 0.3%, the highest reported brightness and efficiency among red, Pb‐free PeLEDs. To avoid oxidation, we identify the chemical pathway of Sn oxidation in 2D‐perovskite films and the formation of SnI_4_ is a key intermediate step. We then stabilized the 2D Sn‐perovskite structure by incorporating HPA into the precursor solution, reducing thereby the trap density and improving the PLQY. HPA is distinguished from other reducing agents in that it forms intermediate complexes during film formation, simultaneously hindering oxidation by inhibiting the formation of SnI_4_ and improving the film quality by seeding the crystal growth. Taken together, the lower Sn^4+^/Sn^2+^ ratio observed in XPS measurements, the weaker bleach intensity in TA pumped at 650 nm, and the longer PL lifetime enabled us to conclude that the Sn^4+^ impurity concentration was decreased when HPA was added. This new class of solution‐processed tin halide PeLEDs represents a promising technology for ultrahigh‐definition displays. The simple yet effective method of incorporating HPA during film formation provides guidelines for more broadly addressing the oxidation of Sn‐based perovskites to enhance performance in both solar cells and PeLEDs.

## Experimental Section

##### Materials

Chemicals listed below are commercially available and used without further purification. Tin (II) iodide (SnI_2_, 99.999%) was purchased from Alfa Aesar. PEAI, hypophosphorous acid solution (H_3_PO_2_, 50 wt% in H_2_O), PHM, toluene (anhydrous, 99.8%), dimethylformamide (DMF) (anhydrous, 99.8%), DMSO (anhydrous, ≥99.9%), and lithium fluoride (LiF) were purchased from Sigma‐Aldrich. PEDOT: PSS (AI 4083) was purchased from Heraeus. 1,3,5‐tris(*N*‐phenylbenzimiazole‐2‐yl)benzene (TPBi) was purchased from Lumtec.

##### Characterization

XRD measurements were conducted using a Rigaku MiniFlex – 6G 600 instrument (Bragg‐Brentano geometry) equipped with D/teX Ultrasilicon strip detector and a Cu *K*_α_ radiation source (λ = 1.5406 Å) operating at a voltage of 40 kV and a current of 15 mA. Optical absorption spectra were measured with a Perkin Elmer 950 UV–vis–NIR spectrometer equipped with an integrating sphere for thin‐film measurements. PL spectra and PL‐decay measurements were carried out using a Horiba FluoroLog‐3 spectrofluorometer in reflection geometry under ambient conditions. The sample was excited using monochromated light (375 nm) from a Xenon lamp. The emission was passed through a 500 nm blaze grating monochromator (iHR320) and collected by an infrared photomultiplier tube. TRPL was measured using 76 MHz ultrafast laser system with Ti:Sapphire oscillator and tunable optical parametric oscillator (OPO) (Coherent Verdi V‐10, Mira‐HP, and Mira‐OPO) pump at 561 nm; laser spot is about 1 µm diameter; the PL was filtered by a 568 nm long pass filter. The streak camera was Hamamatsu C5680‐04. For TA measurements, femtosecond laser pulses of a 1030 nm fundamental beam at a 5 kHz repetition rate were produced using a regenerative amplified Yb‐doped potassium gadolinium tungstate (Yb:KGW) laser (PHAROS, Light Conversion). Part of the fundamental beam was used to pump an optical parametric amplifier (ORPHEUS, Light Conversion) to serve as a narrowband pump, while the other part was focused into a sapphire crystal to generate a white‐light supercontinuum probe (400–1000 nm window with various optical filters). Both the pump and probe pulses were directed into a commercial TA spectrometer (Helios, Ultrafast). Delaying the probe pulse relative to the pump provides a time window of up to 8 ns and the time resolution of these experiments was ≈300 fs (estimated by the rise time of signal amplitudes in TA spectra). The nanocrystals were spin coated onto a glass slide for measurement. TGA experiments were conducted using a PerkinElmer Pyris 1 TGA. About a 10 mg of sample was used for each run and the sample was held at 50 °C to equilibrate for 15 min with a heating rate of 10 °C min^−1^ under nitrogen or air. XPS and UPS measurements were conducted in a PHI5500 multitechnique system with a base pressure of ≈10^−9^ torr. The X‐ray radiation is Al *K*_α_ emission and the UV radiation is He *I*_α_ emission. The photoelectron take‐off angle is set to be 75° and 88° for XPS and UPS, respectively. A bias of −15 V is applied to the sample during UPS measurements. Scanning electron microscope (SEM) images were collected in secondary electron mode by a Hitachi SU5000. It was operated at a voltage of 3 kV with spot size of 3 and intensity of 10.

##### Device Fabrication

0.3 m precursor solution was prepared by dissolving stoichiometric PEAI and SnI_2_ and PHM (30 mg mL^−1^) in DMSO/DMF (1:1) mixture by stirring at room temperature. H_3_PO_2_ additive was directly mixed into the precursor, at an optimized concentration of 20 µL per mL. The solution was filter before application. PEDOT:PSS film (≈30 nm) was spin coated onto a prewashed ITO glass substrate at 5000 r.p.m. for 60 s in air, followed by 30 min annealing at 150 °C in a nitrogen filled glove box. Perovskite precursor solution was spin coated on top via a two‐step process, 1000 r.p.m. for 10 s and then 5000 r.p.m. for 60 s. At the last 10 s in the second step, 250 µL toluene was dropped quickly. Then the substrate was annealed at 100 °C for 30 min to form the solid perovskite (≈200 nm). TPBi (≈60 nm) and LiF/Al (1 nm/100 nm) layers were deposited using a thermal evaporation system. The active area of the device is 6.14 mm^2^ as defined by the overlapping area of the ITO and Al electrodes.

##### Device Characterization

The luminance–current density–voltage characteristics were collected using a HP4140B picoammeter. The absolute EL power spectra of the devices were collected using an integrating sphere and an Ocean Optics USB4000 spectrometer by mounting he devices on the wall of the integrating sphere. The EQEs was then calculated using the measured absolute power spectra and the current density.

## Conflict of Interest

The authors declare no conflict of interest.

## Supporting information

Supporting Information
