# Supplementary material for: High Color Purity Lead‐Free Perovskite Light‐Emitting Diodes via Sn Stabilization
Source: Adv Sci (Weinh). 2020 Mar 1;7(8):1903213. doi: 10.1002/advs.201903213 (PMC7175260; doi:10.1002/advs.201903213)
Supplement: Supplementary file 1 — Supporting Information [file ADVS-7-1903213-s001.pdf]

Copyright WILEY-VCH Verlag GmbH & Co. KGaA, 69469 Weinheim, Germany, 2020.

ADVANCED SCIENCE
Open Access

Supporting Information

for *Adv. Sci.,* DOI: 10.1002/advs.201903213

High Color Purity Lead-Free Perovskite Light-Emitting Diodes via Sn Stabilization

Hongyan Liang, Fanglong Yuan, Andrew Johnston, Congcong Gao, Hitarth Choubisa, Yuan Gao, Ya-Kun Wang, Laxmi Kishore Sagar, Bin Sun, Peicheng Li, Golam Bappi, Bin Chen, Jun Li, Yunkun Wang, Yitong Dong, Dongxin Ma, Yunan Gao, Yongchang Liu, Mingjian Yuan, Makhsud I. Saidaminov, Sjoerd Hoogland, Zheng-Hong Lu, and Edward H. Sargent**

Copyright WILEY-VCH Verlag GmbH & Co. KGaA, 69469 Weinheim, Germany, 2016.

Supporting Information

High Colour Purity Lead-free Perovskite Light-emitting Diode via Sn Stabilization

Hongyan Liang, Fanglong Yuan, Andrew Johnston, Congcong Gao, Hitarth Choubisa, Yuan Gao, Ya-Kun Wang, Laxmi Kishore Sagar, Bin Sun, Peicheng Li, Golam Bappi, Bin Chen, Jun Li, Yunkun Wang, Yitong Dong, Dongxin Ma, Yunan Gao, Yongchang Liu, Mingjian Yuan, Makhsud I. Saidaminov, Sjoerd Hoogland, Zheng-Hong Lu and Edward H. Sargent**

These authors contributed equally: H. Y. Liang, F. L. Yuan, A. Johnston, C. C. Gao.

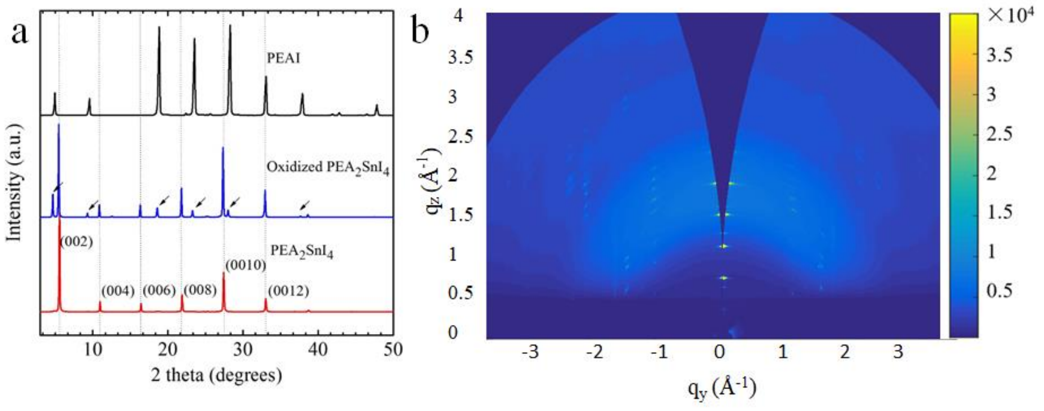

Figure S1. Crystal structure of PEA_2SnI_4. **a** X-ray diffraction (XRD) spectra of films of fresh PEA_2SnI_4,

oxidized PEA_2SnI_4 and PEAI. **b** Grazing-incidence wide-angle X-ray scattering (GIWAXS) pattern of PEA_2SnI_4 films.

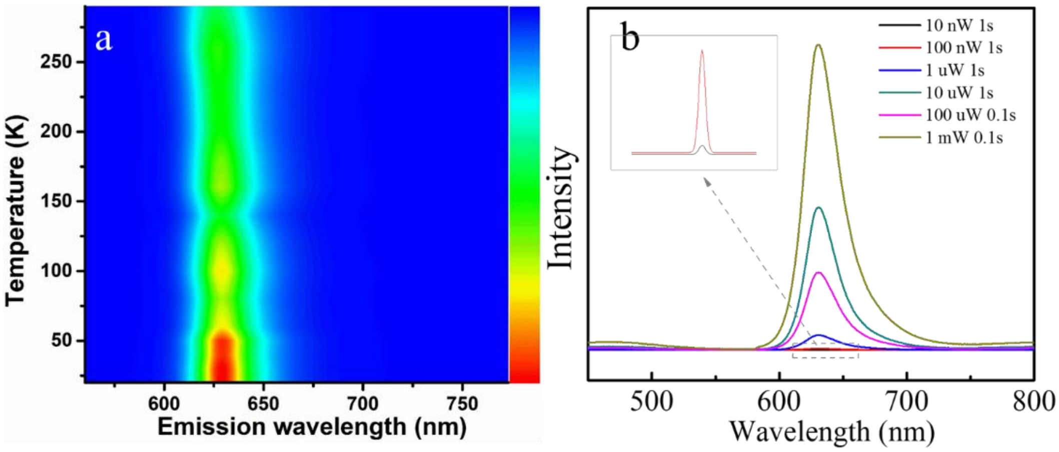

Figure S2. Optical stability of PEA_2SnI_4 perovskite. **a** Temperature dependent and **b** power-dependent photoluminescence. The integrated time for excitation powder lower than 100 μW was 1s and higher than 100 μW was 0.1s.

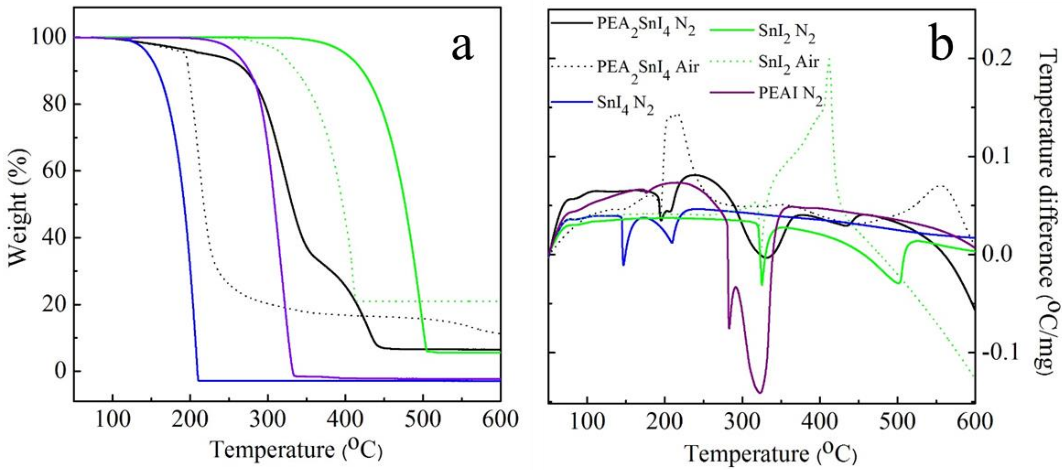

Figure S3. Thermogravimetric analysis (TGA) of PEA_2SnI_4, SnI_4, SnI_2 and PEAI. **a** Relationship between weight loss and **b** rate of weight loss vs temperature. All samples were measured in N_2. TGA measurements of PEA_2SnI_4 and SnI_2 were also performed in air. After 210 °C, the mass of PEA_2SnI_4 in air is 64.9%; the mass loss is due to the evaporation of SnI_4. The second region of mass loss occurs due to the evaporation of PEAI. SnI_2 in air sublimes at 415 °C, with a mass loss of 79.6%.

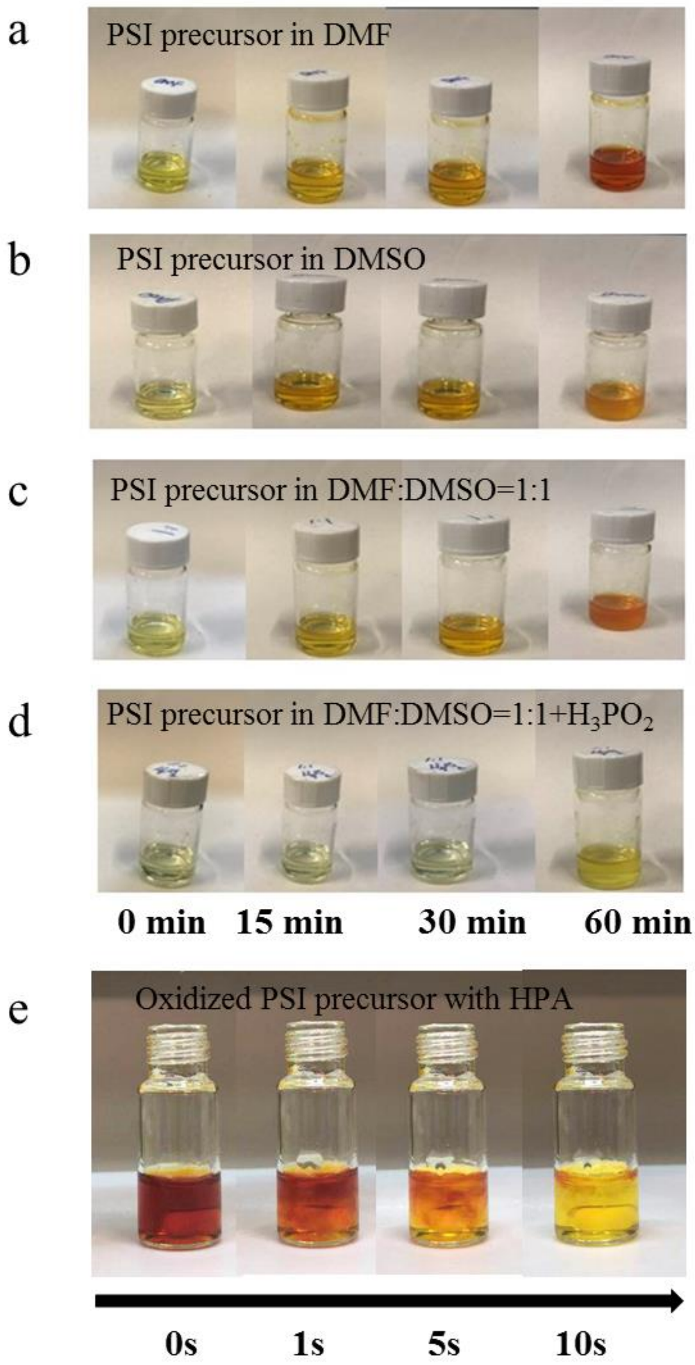

Figure S4. Color change of PEA_2SnI_4 precursor solutions caused by oxidation in different conditions. Photographs of 0.1 mol/L PEA_2SnI_4 in **a** DMSO, **b** DMF, **c** DMSO: DMF = 1:1 and **d** DMSO: DMF = 1:1 with 20 μL/mL H_3PO_2 taken after different exposure times to air. **e** The gradual lightening of an oxidizaed PEA_2SnI_4 precursor solution after HPA additive.

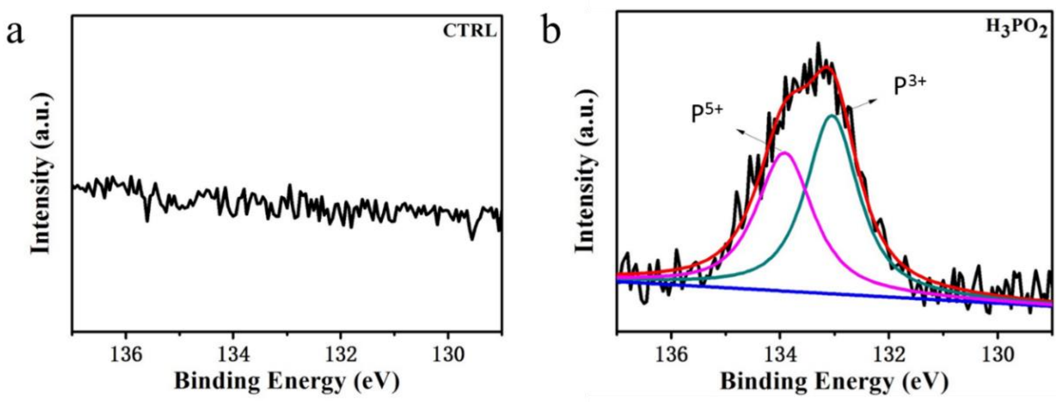

Figure S5. X-ray photoelectron spectroscopy (XPS) measurements. High-resolution P $2p_{3/2}$ core level XPS spectra without **a** or with **b** HPA additive.

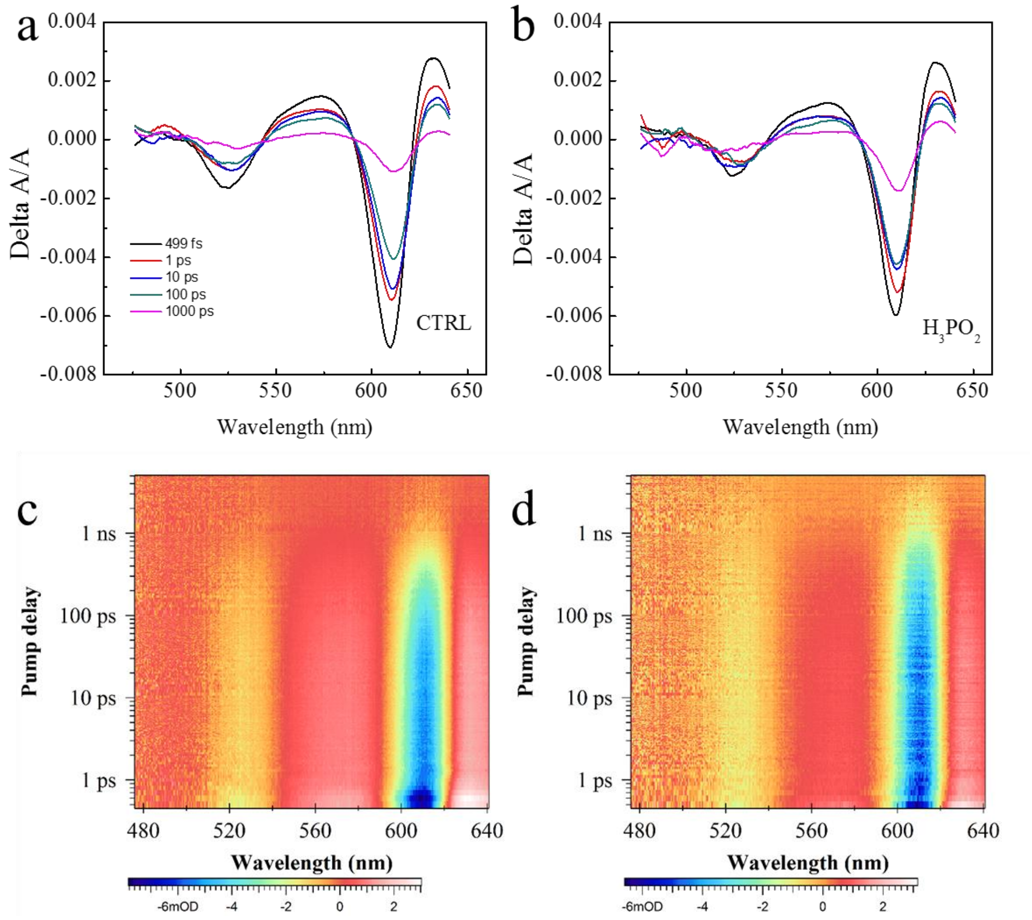

Figure S6. Transient absorption (TA) spectra pump with 400 nm laser **a**, **b** TA spectra at selected delay times of perovskite without and with H_3PO_2 additive. **c, d** Pseudo-color TA plot without and with H_3PO_2 additive.

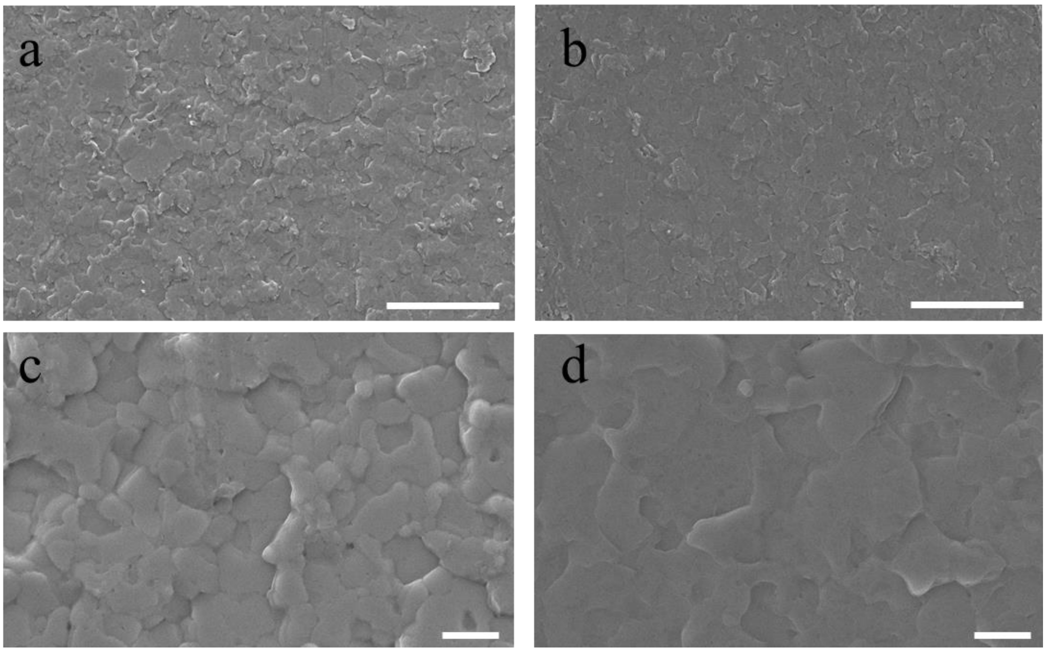

Figure S7. SEM images of PEA_2SnI_4 film. **a**, **c** without and **b**, **d** with H_3PO_2 additive at different magnifications. The scale bars in **a, b** are 10 μm and in **c, d** are 1μm.

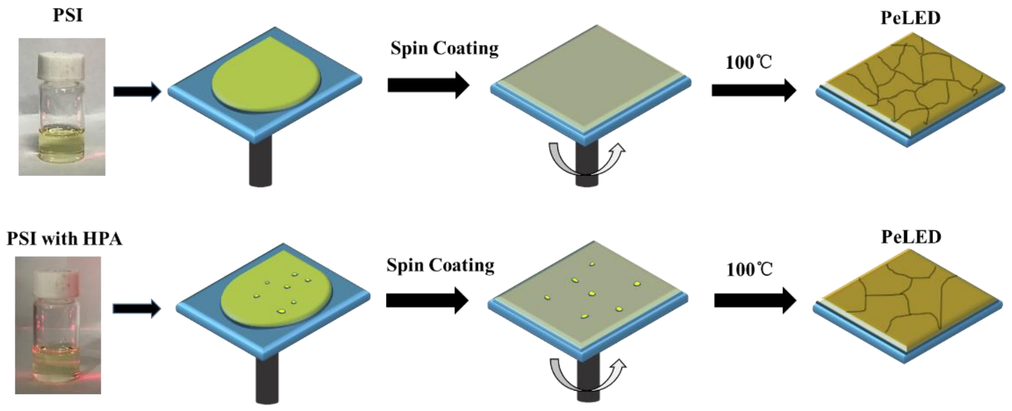

$$2H_2PO_2^- + SnI_2 \longrightarrow Sn(H_2PO_2)_2 + 2I^- \downarrow$$

Figure S8. Schematic illustration of crystallization mechanism during film fabrication process w/wo HPA. With HPA additive, the Tyndall effect is observed which confirms the colloid formation.

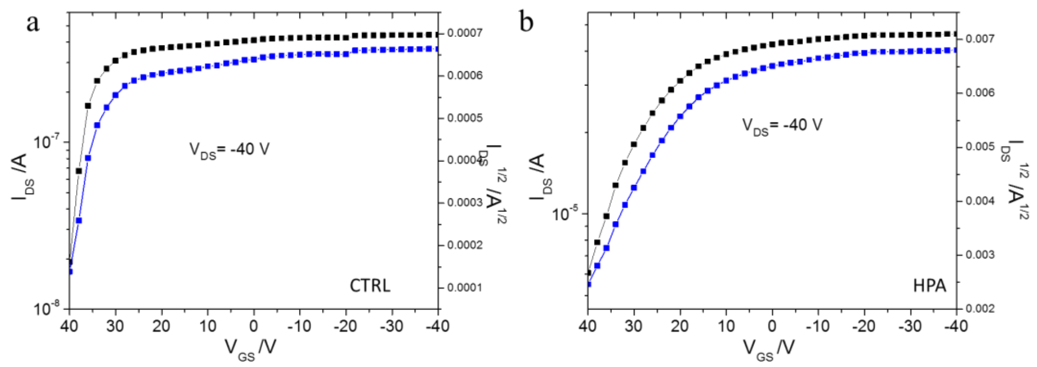

Figure S9. Representative output and transfer characteristics of transistor without/with HPA additive. The mobility is **a** ~ 0.007 cm^{2}v^{-1}s^{-1} for the control sample and **b** ~ 0.07 cm^{2}v^{-1}s^{-1} for the sample with the HPA additive.

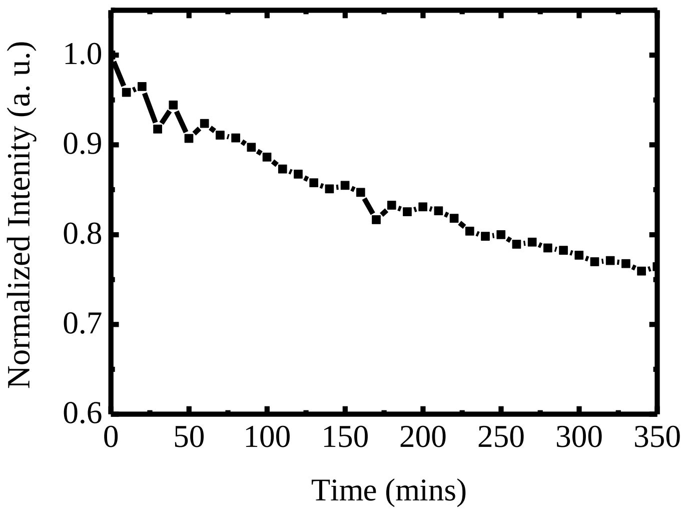

Figure S10**.** The optical stability of PSI film under continuous light.

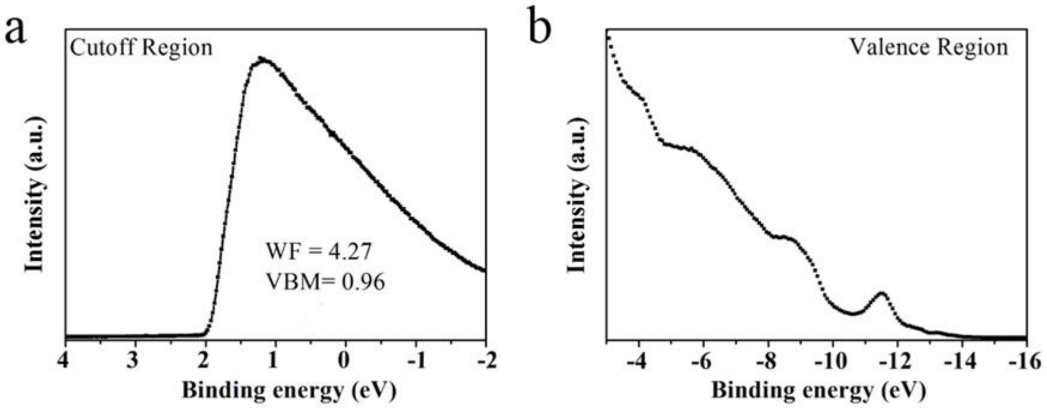

Figure S11. Ultra-violet photoelectron spectroscopy (UPS) of the PEA_2SnI_4 perovskite film on an ITO glass substrate. UPS was used to determine the valence band minima (VBM). Conduction band minima (CBM) was calculated based on the optical band gaps. The energy levels are referenced to the vacuum level.

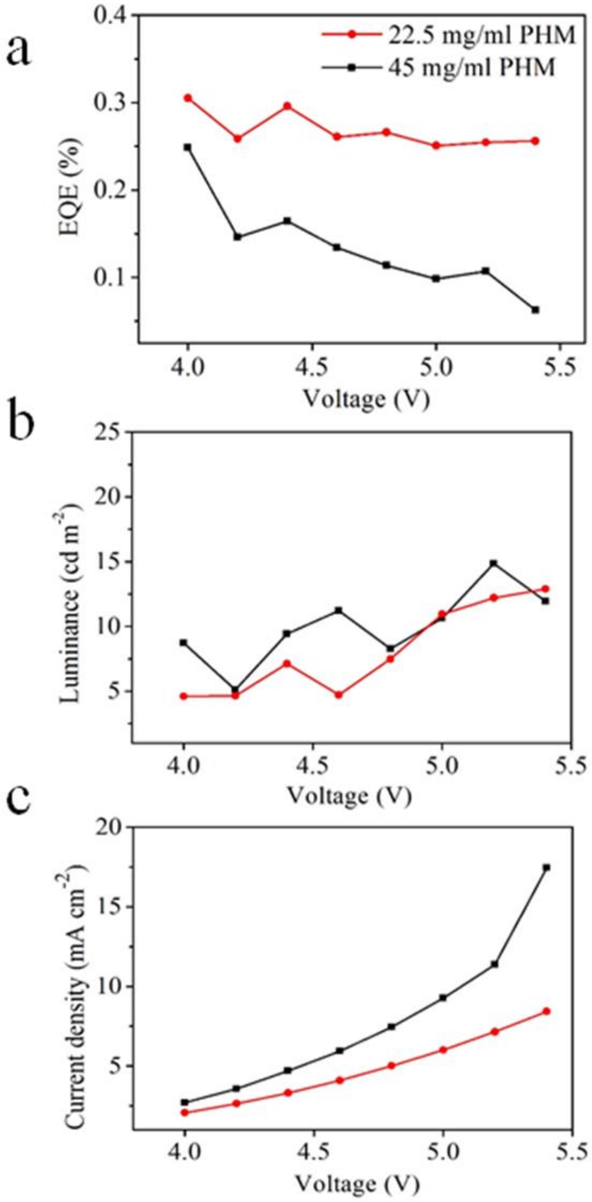

Figure S12. Optoelectronic characteristics of PeLEDs with different amount of PHM additive. Dependence of **a** EQE, **b** luminance and **c** the current density on driving voltage.

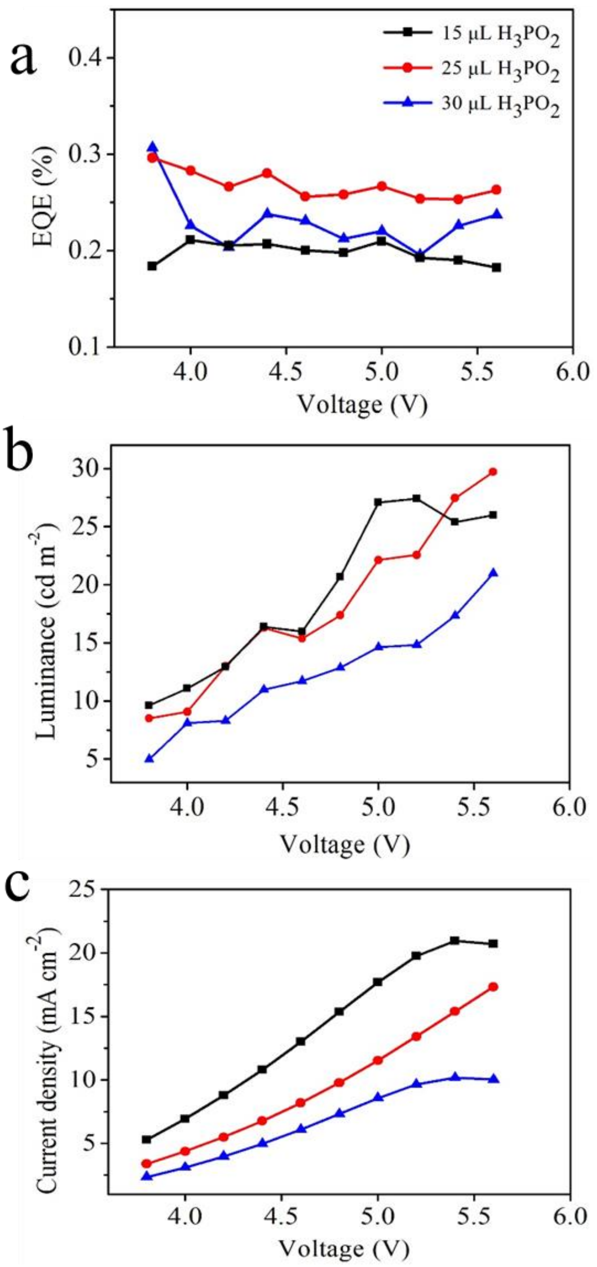

Figure S13. Optoelectronic characteristics of PeLEDs with different amount of HPA additive. Dependence of **a** EQE, **b** luminance and **c** the current density on driving voltage.

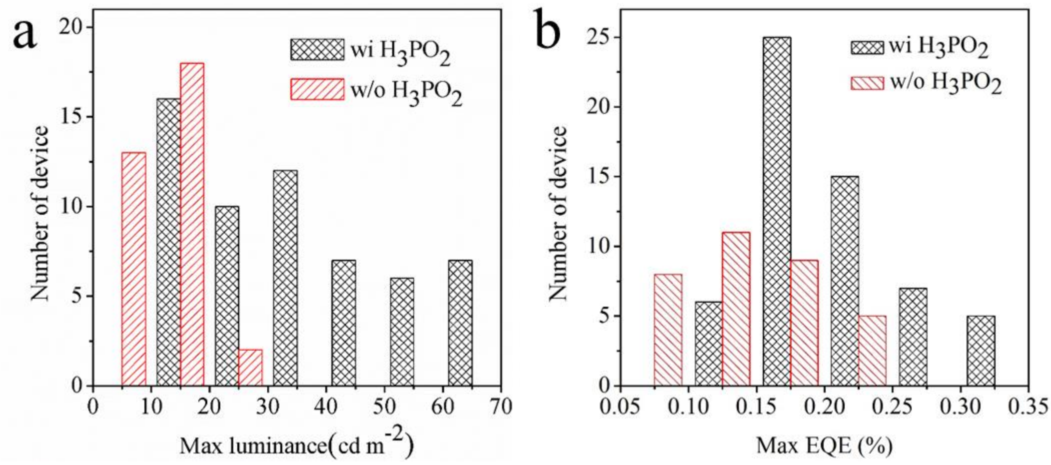

Figure S14. Statistical data of device performance. **a** Histograms of maximum luminance and **b** maximum EQE measured from 58 devices with H_3PO_2 and 33 devices without H_3PO_2.

Table S1: Lifetime shown in Fig. 3f

	A1	t_1 (ns)	A2	t_2 (ns)	T (ns)
**CTRL**	0.367	0.498	0.633	0.122	0.386
**H_3PO_2**	0.413	1.334	0.587	0.662	1.056

Table S2: Summary of color purity of red PeLEDs

REF	Materials	Peak (nm)	FWHM (nm)	CIE x,y	EQE (%)	L ($cd\ m^{-2}$)
**[1]**	$CsPb(Br/I)_3$ with OAM-I	653	33	0.72, 0.28	21.3%	500
	$CsPb(Br/I)_3$ with An-HI	645	30	0.71, 0.28	14.1%	794
**[2]**	$CH_3NH_3PbI_2Br$	635	43	-	2.75%	-
**[3]**	$CsPbI_3$	678	32	0.67, 0.26	5.92%	1250
**[4]**	$PBA_2Cs_{n-1}Pb_nI_{3n+1}$	664	-	0.72, 0.27	13.3%	968
**[5]**	$(BA)_2(Cs)_{n-1}[Pb_nI_{3n+1}]$	680	39	0.71, 0.28	6.23%	1392
**[6]**	$CsSnBr_3$	672	54	-	0.34%	172
**[7]**	$(OAm)_2SnBr_4$	621	163	-	0.1%	350
**[8]**	PEA_2SnI_4	618	-	-	-	0.15
**This work**	**PEA_2SnI_4**	**633**	**24**	**0.706, 0.294**	**0.3%**	**70**
**Rec.**		**630**	**0**	**0.708, 0.292**		

2020

Note S1: To compare if H_3PO_2 is more readily oxidized than Sn^{2+}, density functional theory (DFT) calculations were done using **VASPsol**[9,10] which implements an implicit solvation model. We modeled the two oxidation processes with the following reactions:

Reaction 1: DMSO + Sn^{2+} + $2H^+$ -> DMS + Sn^{4+} + H_2O

Reaction 2: H_3PO_2 + DMSO -> H_3PO_3 + DMS

Using the dielectric constant of DMSO (46.7) and a uniform k-points grid of 2-2-2, we get a total energy change of +30 eV for oxidation of Sn^{2+} and a total energy change of -0.576 eV for oxidation of H_3PO_2, indicating preferential oxidation of H_3PO_2 compared to Sn^{2+}.

References

[1] T. Chiba, Y. Hayashi, H. Ebe, K. Hoshi, J. Sato, S. Sato, Y. Pu, S. Ohisa, J. Kido, *Nat. Photonics* **2018**, *12*, 681-687.

[2] Y. Hassan, O. J. Ashton, J. H. Park, G. Li, N. Sakai, B. Wenger, A. A. Haghighirad, N. K. Noel, M. Song, B. R. Lee, R. H. Friend, H. J. Snaith, *J. Am. Chem. Soc.* **2019**, *141*, 1269-1279.

[3] J. Yao, J, Ge, K, Wang, G. Zhang, B. Zhu, C. Chen, Q. Zhang, Y. Luo, S. Yu, H. Yao, *J. Am. Chem. Soc.* **2019**, *141*, 2069-2079.

[4] Z. He, Y. Liu, Z. Yang, J. Li, J. Cui, D. Chen, Z. Fang, H. He, Z. Ye, H. Zhu, N. Wang, J. Wang, Y. Jin, *ACS Photonics* **2019**, *6*, 587-594.

[5] Y. Tian, C. Zhou, M. Worku, X. Wang, Y. Ling, H. Gao, Y. Zhou, Y. Miao, J. Guan, B. Ma, *Adv. Mater.* **2018**, *30*, 1707093.

[6] F. Yuan, J. Xi, H. Dong, K. Xi, W. Zhang, C. Ran, B. Jiao, X. Hou, A. K. Y. Jen, Z. Wu, *Phys. Status. Solidi-R* **2018**, *12*, 1800090.

[7] X. Zhang, C. Wang , Y. Zhang, X. Zhang, S. Wang, M. Lu, H. Cui, S. V. Kershaw, W. Yu, A. L. Rogach, *ACS Energy Lett.* **2019**, *4*, 242.

[8] L. Lanzetta, J. Marin-Beloqui, I. Sanchez-Molina, D. Ding, S. A. Haque, *ACS Energy Lett.* **2017**, *2*, 1662-1668.

[9] K. Mathew, R. Sundararaman, K. Letchworth-Weaver, T. A. Arias, R. G. Hennig, *J. Chem. Phys.* **2014**, *140*, 084106.

[10] K. Mathew, R. G. Hennig, *arXiv preprint arXiv* **2016**, *1601*, 03346.
